# Supplementary material for: Evidence for social parasitism of early insect societies by Cretaceous rove beetles
Source: Nat Commun. 2016 Dec 8;7:13658. doi: 10.1038/ncomms13658 (PMC5155144; doi:10.1038/ncomms13658)
Supplement: Supplementary Information — Supplementary Figures 1-3, Supplementary Tables 1 and 2, Supplementary Note 1 and Supplementary References [file ncomms13658-s1.pdf]

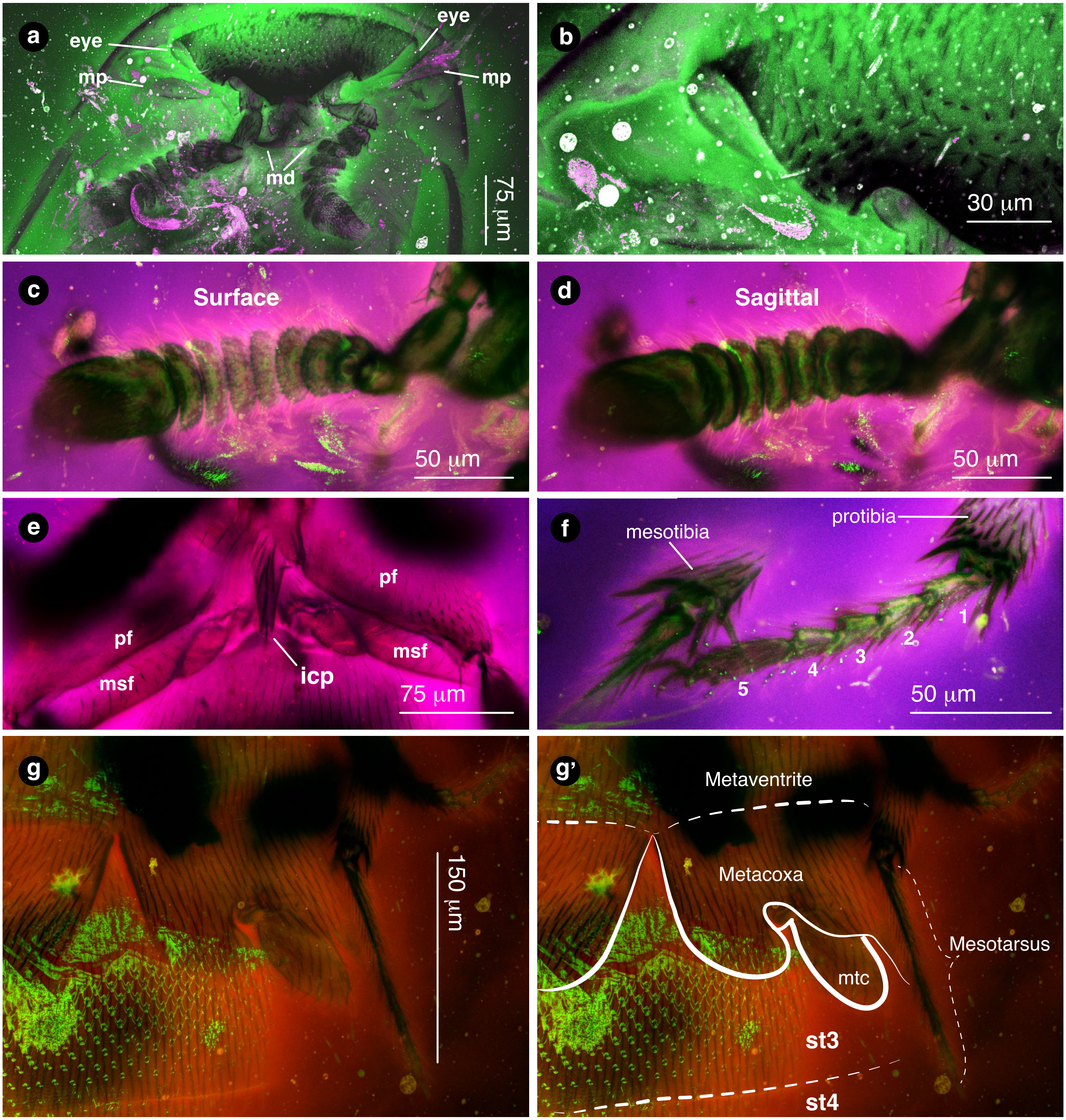

Supplementary Figure 1. Confocal reconstructions of key morphological features of *Mesosymbion*.
a: Triangular, opisthognathous head shielded by limuloid pronotum, showing position of narrow eyes extending from apices along edges of vertex; md: mandibles, mp: maxillary palpus. **b**: Enlarged image of right eye. **c, d**: Left antenna, confocal plane on antennal surface (**c**) and through a sagittal plane (**d**), showing telescoping of compact antennomeres and reduction/loss or concealment of interconnecting pedicels. **e**: Mesocoxal area, showing long, pointed intercoxal process (icp). **f**: Protarsus, with 5 tarsomeres indicated. **g, g'**: Metacoxa with lamella partially covering metatrochanter (mtc); st: sternite. The latticed body sculpturation is also evident in these images.

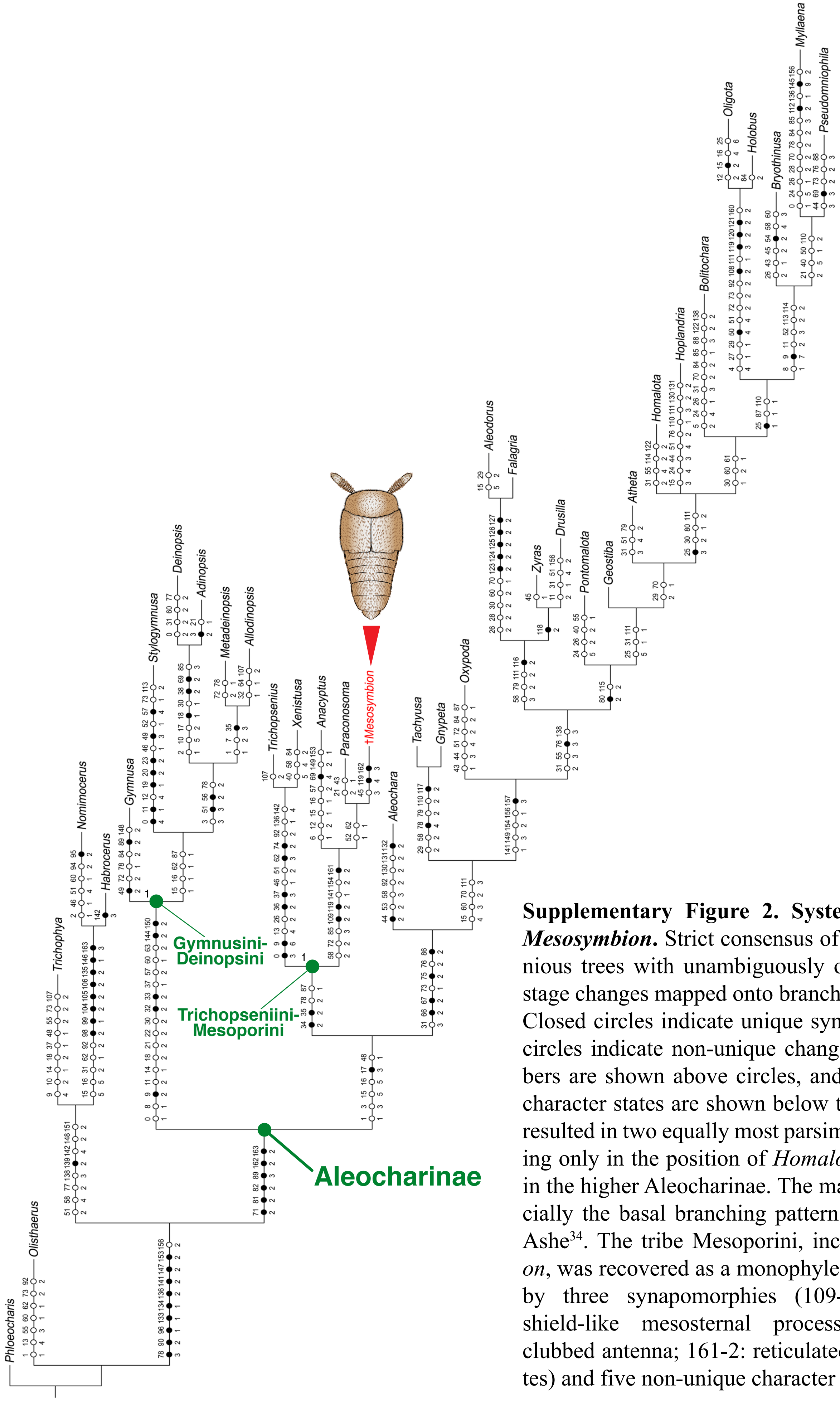

Supplementary Figure 2. Systematic position of *Mesosymbion*. Strict consensus of two most parsimonious trees with unambiguously optimized character stage changes mapped onto branches using WinClada. Closed circles indicate unique synapomorphies, open circles indicate non-unique changes. Character numbers are shown above circles, and the corresponding character states are shown below them. TNT analysis resulted in two equally most parsimonious trees differing only in the position of *Homalota* and *Hoplandria* in the higher Aleocharinae. The major topology, especially the basal branching pattern, is congruent with Ashe[34]. The tribe Mesoporini, including *Mesosymbion*, was recovered as a monophyletic group supported by three synapomorphies (109-2: expanded and shield-like mesosternal process; 119-2: weakly clubbed antenna; 161-2: reticulated abdominal sternites) and five non-unique character states.

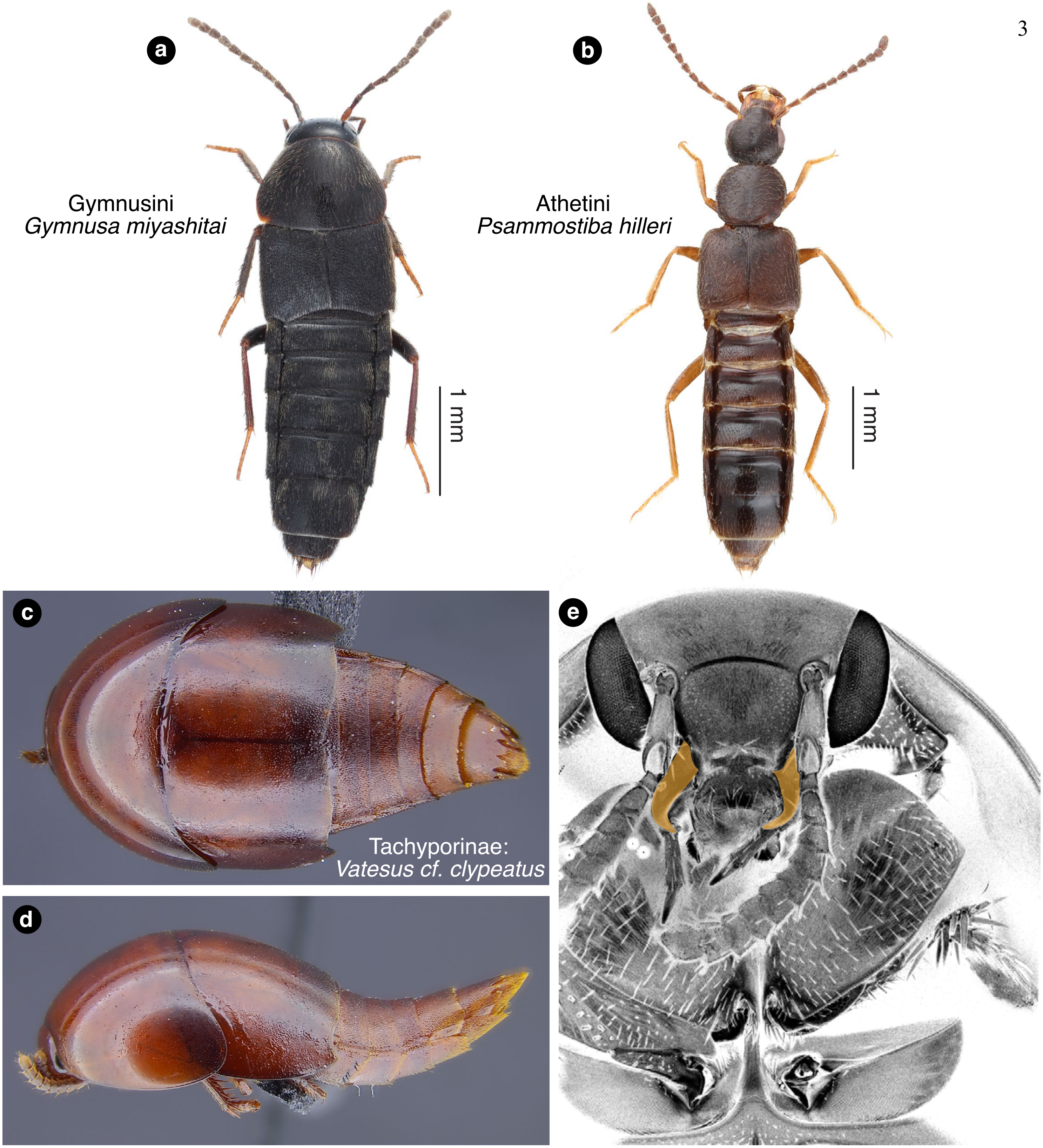

Supplementary Figure 3. Comparative aleocharine anatomy and convergent limuloid morphology of *Vatesus* (Tachyporinae). a: *Gymnusa miyashitai* (Gymnusini) with quasi-limuloid morphology, filiform antennae and prognathous/weakly hypognathus head orientation in the earliest-diverging clade of Aleocharinae **b:** Generalized higher aleocharine, *Psammostiba hilleri* (Athetini), with non-limuloid morphology, filiform antennae and prognathous head orientation. **c, d**: Habitus of *Vatesus cf. clypeatus* (Costa Rica, ant host: *Eciton hamatum*) showing fully limuloid morphology with exaggerated expansion of the pronotum. Images courtesy of Christoph von Beeren[40]. **e**: Confocal reconstruction of *Vatesus* head in ventral view, showing triangular shape, opisthognathous orientation, compact antennae with concealed/absent pedicels and falciform mandibles (highlighted orange) pointing caudally.

Supplementary Table 1. Taxonomic inventory and provenance of described fossil Aleocharinae. Quaternary species are not included. Tribes in bold belong to the higher Aleocharinae. The asterisk next to *Homalota recisa* signifies an incorrect genus assignment: at that time of original description, the genus *Homalota* was equivalent to what is now *Atheta* (Athetini). By 1900 the concept had changed (because of type species designations) and *Homalota* s.str. became used in a different, very restricted sense, while nearly all older "*Homalota*" were transferred to *Atheta*. However, nobody has officially transferred *H. recisa* to *Atheta*, and therefore the fossil is here listed as a *Homalota* species.

**Taxon**	**Tribe**	**Chronostratigraphy**	**Deposit**
Oxypoda bindosa Seevers[1]	**Oxypodini**	Miocene	Mexican amber
Ambracyptus minutissima (Seevers)[1,2]	Mesoporini	Miocene	Mexican amber
Prorhinopsenius mexicanus (Seevers)[1]	Trichopseniini	Miocene	Mexican amber
Prorhinopsenius alzadae Kistner[3]	Trichopseniini	Miocene-Oligocene	Dominican amber
Prorhinopsenius poinari Kistner[3]	Trichopseniini	Miocene-Oligocene	Dominican amber
Prorhinopsenius seeversi Kistner[3]	Trichopseniini	Miocene-Oligocene	Dominican amber
Hygronoma deleta Oustalet[4]	**Hygronomini**	Oligocene	Niveau du gypse d'Aix
Leptusa (*Protoleptusa*) *defuncta* Semenov *et al*[5]	**Homalotini**	Oligocene-Eocene	Rovno amber
Adinopsis groehni Zerche[6]	Deinopsini	Eocene	Baltic amber
Aleochara (*Aleochara*) *baltica* Paśnik & Kubisz[7]	**Aleocharini**	Eocene	Baltic amber
Atheta (*Datomicra*) *jantarica* Paśnik[8]	**Athetini**	Eocene	Baltic amber
Baltioligota electrica Paśnik[8]	**Hypocyphtini**	Eocene	Baltic amber
Dictyon antiquus Paśnik & Kubisz[7]	Mesoporini	Eocene	Baltic amber
Electrogymnusa baltica Wolf-Schwenniger[9]	Gymnusini	Eocene	Baltic amber
Palaeomesoporus electricus Yamamoto & Maruyama[10]	Mesoporini	Eocene	Baltic amber
Phymatura electrica Paśnik & Kubisz[7]	**Homalotini**	Eocene	Baltic amber
Atheta florissantensis Wickham[11]	**Athetini**	Eocene	Florissant
Gyrophaena saxicola Scudder[12]	**Homalotini**	Eocene	Green River
Homalota recisa Scudder[13]*	**Homalotini**	Eocene	Green River
Cretodeinopsis aenigmatica Cai & Huang[14]	Deinopsini	Cretaceous (Cenomanian)	Burmese amber
Mesosymbion compactus (this study)	Mesoporini	Cretaceous (Cenomanian)	Burmese amber

Supplementary Table 2. Character Matrix for phylogenetic analysis. Characters 0–163 and states for each taxon are from Ashe[15], where the original matrix and full taxon names can be found. The matrix below can also be found in the MrBayes nexus file, Supplementary Data 1.

```
                                    1         2         3         4
                          0         0         0         0         0
Phloeocharis_subtill.     22315112211312344232111124?21111111112121112112221
Olisthaerus_substriatus   21215132211314333132111125321111111112121111112121
Trichophya_texana         2221313124231213312211?112321211111111121111112 21?
Nomimocerus_marginatus    22115132211312355132111124321112111112121111111121
Habrocerus_capill.        22215131211312355132111122321212111112121111112121
Gymnusa_variegata         1231213112423313312212214631?121223111122222132122
Stylogymnusa_subantarct.  4221??3112414411112122223611?121????11121222111123
Metadeinopsis_brunnea     1123223215423411112212211531?121223311113112132121
Allodinopsis_howdeni      11234?3215423411112212211531?121123311112112132121
Deinopsis_erosa           22134?3114523411121212211611?112221111214112132121
Adinopsis_sp.             1212313114523411121211211511?111221111226112132121
Paraconosoma_naviculare   2121333213423245523212114632111111221212111111221?
Anacyptus_testacaeus      2133311221232231133211111532121111221212111211221?
Trichopsenius_depressus   31334?3226533435533211112421121111222312111211121?
Xenistusa_hexagonalis     31334?3226533435533211112421121111222312511211121?
Myllaena_gracili.         11333?3217323234533212215112221111111212511213221?
Pseudomniophila_sp.       2133311217323234533212113132421111111212511233221?
Bryothinusa_catalina      2133311217324434533211213122421111111212111112221?
Atheta_crassicornis       2133313221131234533211113532323311111212111211221?
Geostiba_cicellaris       2133312221131234533211113532113111111212111212221?
Pontomalota_opaca         2133313221131234533211115422113211111212211213221?
Zyras_haworthi            2133313221131234533211113432113211111212111211221?
Drusilla_canaliculata     2133313221121234533211113432113111111212111212221?
Oligota_atomaria          21334?3221232432433211113631?11111111212111213221?
Holobus_flavicornis       21334?3221231434533211113131?11111111212111213221?
Oxypoda_longipes          2133311221131234533211113432113311111212111133221?
Tachyusa_sp.              2133312221131234533211113432321311111212111212221?
Bolitochara_obliqua       2133322221131234533211114312421311111212111213221?
Aleodorus_bilobatus       2133312221131235533211113422222211111212111212221?
Falagria_dissecta         2133312221131234533211113422212211111212111212221?
Homalota_plana            2133312221131234533211113332422211111212111211221?
Aleochara_curtula         21333112211312355332111156 31?133111112121112212 21?
Hoplandria_lateralis      2133311221131233533211114332322111111212111233221?
Gnypeta_carbonaria        2133312221131234533211113432221311111212111212221?
Mesosymbion_compactus     2??????????????55??2?11???????????????????1213221?


                          5         6         7         8         9
                          0         0         0         0         0
Phloeocharis_subtill.     142?1411212122?111111113111111111??112111111212112
Olisthaerus_substriatus   11311311111111?111111112111111111??112111?21112112
Trichophya_texana         123?1211412221?111111112111231111??112112111111112
Nomimocerus_marginatus    142?1411411211?1???????31112?11????????12121221121
Habrocerus_capill.        122?1411412211?1???????31112?11????????12121111121
Gymnusa_variegata         11311312111222111111122311111112212112122111111112
Stylogymnusa_subantarct.  111???1411??12211111121111113112233211132111111112
Metadeinopsis_brunnea     1332112211121221??11122311111112221211132?11111112
Allodinopsis_howdeni      1332112211??1211??11121311112112211?11132?11111112
Deinopsis_erosa           5332112211221221??12121311122112221311132111111112
Adinopsis_sp.             6332112211121221??12121311112112221311132111111112
Paraconosoma_naviculare   111???11212111?11121122311112112211311132?11111112
Anacyptus_testacaeus      112?1412212121?11124122311?12112211311132?11111112
```

```
Trichopsenius_depressus  122?1411112131?1??11121321112112212111?32?21111112
Xenistusa_hexagonalis    122?1411412131?1??11121321112112212211?32?21111112
Myllaena_gracili.        11311213112121?12211221112312112222321232?11111112
Pseudomniophila_sp.      11311212312121?12213121212213112223221332?11111112
Bryothinusa_catalina     31312111413121?12211121112313112223221232?11111112
Atheta_crassicornis      242?1311113221?12211121112313222223222232?11111112
Geostiba_cicellaris      212?1311113221?12211221112313122223222232?11111112
Pontomalota_opaca        212?1111113121?12211221112313122223222232?11111112
Zyras_haworthi           212?1311313221?12211221112313212223222232?11111112
Drusilla_canaliculata    242?1311313221?12211221112313?12223222232?11111112
Oligota_atomaria         442?1311312121?12211122212313112223221232?21111112
Holobus_flavicornis      442?1311312121?12211122212313112222221232?21111112
Oxypoda_longipes         142?1411113121?12211222112213112222221132?11111112
Tachyusa_sp.             211???11213121?12211221112214212213222332?11111112
Bolitochara_obliqua      312?1311212121?12211221112313112222122332?11111112
Aleodorus_bilobatus      212?1311312121?12211121112313212223222232?11111112
Falagria_dissecta        212?1311312121?12211121112313212223222232?11111112
Homalota_plana           312?1411113221?12211121112313112223222232?11111112
Aleochara_curtula        11321411312221?12211121112213112223222332?21111112
Hoplandria_lateralis     242?1311313221?12211121112213112223222232?11111112
Gnypeta_carbonaria       211???11213221?12211221112214212213222332?11111112
Mesosymbion_compactus    111???112???11??1?111??31???????????????2?11??????

                         1         1         1         1         1
                         0         1         2         3         4
                         0         0         0         0         0
Phloeocharis_subtill.    1232111111111111111111111111?111122111211111142111
Olisthaerus_substriatus  1231111111111111111111111111?111122111211111142111
Trichophya_texana        1231111211111111111111111111?11111112?321242132223
Nomimocerus_marginatus   1231222111111111111111111111??1111122?321243121222
Habrocerus_capill.       1231222111111111111111111111??1111122?321233121222
Gymnusa_variegata        1231111111111111111111111111?11111112?212211282221
Stylogymnusa_subantarct. 12321111111112?1111111111111?1111?????????????????
Metadeinopsis_brunnea    1231111111111111111111111111?1111?????????????????
Allodinopsis_howdeni     1232111211111111111111111111?1111?????????????????
Deinopsis_erosa          1231111111111111111111111111?11111112?212211282211
Adinopsis_sp.            1232111111111111111111111111?1111?????????????????
Paraconosoma_naviculare  1232111112111111111211111111?11111112?2121111?2211
Anacyptus_testacaeus     123?111112111111111211111111?11111112?212111112212
Trichopsenius_depressus  123?1112111111111111111111111?11111111??11241?12211
Xenistusa_hexagonalis    123?111111111111111111111111?111111112311241112211
Myllaena_gracili.        123?111111222223111111111111?111111111312111192213
Pseudomniophila_sp.      123?111111221222111111111111?11111112?312111112213
Bryothinusa_catalina     123?111111121221111111111111?1111?????????????????
Atheta_crassicornis      123?111111311112111111111111?11111112?312111112213
Geostiba_cicellaris      123?111111311112111111111111?1111?????????????????
Pontomalota_opaca        123?111111331112111111111111?1111?????????????????
Zyras_haworthi           123?111111121111212111111111?11111112?312111112223
Drusilla_canaliculata    123?111111121111212111111111?11111112?312111112223
Oligota_atomaria         123?111121111111111322111111?11111112??121111122?3
Holobus_flavicornis      123?111121111111111322111111?1111?????????????????
Oxypoda_longipes         123?111111131111111111111111?111111121212111112213
Tachyusa_sp.             123?111111231111121111111111?111111112?31211111221
Bolitochara_obliqua      123?111111321112111111211111?11111112?212111112213
Aleodorus_bilobatus      123?11111132111211111112222221111?????????????????
Falagria_dissecta        123?11111122111211111112222221111?????????????????
Homalota_plana           123?111111321122111111211111?1111?????????????????
Aleochara_curtula        123?111111311111111111111111?1222?????????????????
Hoplandria_lateralis     123?111111131112111111111111?1221?????????????????
Gnypeta_carbonaria       123?111111231111121111111111?1111?????????????????
Mesosymbion_compactus    1?3?11?112??1??11??41?????1????1??????????????????
```

```
                         1         1
                         5         6
                         0         0
Phloeocharis_subtill.    11111211?1111
Olisthaerus_substriatus  11111211111111
Trichophya_texana        12221222111111
Nomimocerus_marginatus   12121221111113
Habrocerus_capill.       12121222111113
Gymnusa_variegata        21221221111122
Stylogymnusa_subantarct. ??????????1122
Metadeinopsis_brunnea    ??????????1122
Allodinopsis_howdeni     ??????????1122
Deinopsis_erosa          22221221111122
Adinopsis_sp.            ??????????1122
Paraconosoma_naviculare  1122222112?22
Anacyptus_testacaeus     11212222112222
Trichopsenius_depressus  112212??312122
Xenistusa_hexagonalis    112212??311122
Myllaena_gracili.        11212223221122
Pseudomniophila_sp.      11212213221122
Bryothinusa_catalina     ??????????1122
Atheta_crassicornis      11212213221122
Geostiba_cicellaris      ??????????1122
Pontomalota_opaca        ??????????1122
Zyras_haworthi           11222213221122
Drusilla_canaliculata    11222223221122
Oligota_atomaria         112?22?2222122
Holobus_flavicornis      ??????????2122
Oxypoda_longipes         11222213221122
Tachyusa_sp.             31121221321122
Bolitochara_obliqua      11212213221122
Aleodorus_bilobatus      ??????????1122
Falagria_dissecta        ??????????1122
Homalota_plana           ??????????1122
Aleochara_curtula        ??????????1122
Hoplandria_lateralis     ??????????1122
Gnypeta_carbonaria       ??????????1122
Mesosymbion_compactus    ??????????223?
```

Supplementary Note 1

Systematic Palaeontology

Order Coleoptera Linnaeus, 1758

Superfamily Staphylinoidea Latreille, 1802

Family Staphylinidae Latreille, 1802

Subfamily Aleocharinae Fleming, 1821

Tribe Mesoporini Cameron, 1959

Genus *Mesosymbion* gen. nov.

ZooBank ID: http://zoobank.org/urn:lsid:zoobank.org:act:43DA781F-068A-4578-B428-5327DE78EC21

Type species: *Mesosymbion compactus* sp. nov. here designated

***Diagnostic Description*:** Body small (1.04 mm), limuloid, broad and relatively flattened, with short, strongly tapering abdomen (Figs 1a–c, Supplementary Videos 1, 2). Surface densely covered with short microsetae (Figs 1b, c, Supplementary Videos 1, 2). Head opisthognathous, completely concealed under pronotum, no part of it visible in dorsal view (Figs 1a–d, Supplementary Fig 1a, Supplementary Video 4, but see Fig 3b for possible articulation of extended occiput beyond anterior margin of pronotum). Antennae clavate, short and broad (concealed underneath pronotum in the holotype specimen) with transverse antennomeres compacted together with concealed/absent pedicels (Figs 1f, g, Supplementary Figs 1a, c, d, Supplementary Video 3). Mandibles slender, falciform, lacking apical inner teeth, their bases seemingly contiguous or close together (Fig 1d, Supplementary Fig 1a, Supplementary Video 4). Maxillary palpomere 4-segmented (Fig 1e, Supplementary Video 4); palpomere 3 long, slender, likely as long as palpomere 2 (basal half not visible) (Fig 1e); palpomere 4 (last), small, conical, much narrower than palpomere 3, less than half length of penultimate palpomere (Fig 1e, Supplementary Video 4). Pronotum transversely oval (Fig 1b), with arcuate anterior and posterior margins, lacking macrosetae. Elytra widest at apical third (Figs 1a–c, Supplementary Videos 1, 2), slightly longer than pronotum; posterolateral margins of elytra strongly

emarginate (Fig 1b, Supplementary Video 1). All tarsi 5-segmented (Supplementary Fig 1f, Supplementary Videos 1, 2); mesotarsi slightly longer than mesotibiae (Fig 1c, Supplementary Video 2); metatarsi moderately longer than metatibiae (Figs 1a, b, Supplementary Videos 1, 2). Hindcoxa with moderately developed coxal lamella (Figs S1g, g', Supplementary Video 5), with posterior margin only partially covering the coxa itself. Abdomen lacking prominent macrosetae (Figs 1a–c, Supplementary Video S2); abdominal segments III–VII each with a pair of paratergites (Figs 1a, b, Supplementary Video 1).

***Mesosymbion compactus* sp. nov.**

ZooBank ID: http://zoobank.org/urn:lsid:zoobank.org:act:6D007E14-D12B-434E-937E-61D77A11CB35

Holotype Material: Sex unknown (putative male). Data label: "AMBER: MYANMAR (BURMA), Upper Cretaceous, Kachin: Noije Bum mines, near Tanai Village (105 km NW Myitkyina), AMNH Bu-SY5". Specimen in AMNH.

Diagnosis. As for genus.

Description. Body length 1.04 mm (from base of head capsule to apex of abdomen), overall form compact and broad (body length 2.1 × longer than width across elytra). Body shape limuloid (horse-shoe crab- or teardrop-shaped) with enlarged pronotum fully covering head in dorsal view (Figs 1a–d, Supplementary Fig 1a, Supplementary Video 4). Body weakly convex and strongly tapered posteriorly (Figs 1a–c, Supplementary Videos 1, 2). Surface covered with prominent latticed reticulation (Fig 1c, Supplementary Figs 1g–g'). Dorsal and ventral surfaces uniformly densely pubescent with posteriorly-directed setae (Figs 1b, c, Supplementary Videos S1, 2); lacking longer macrosetae. Body color uniformly dark brown (Fig 1a); mouthparts, legs, posterior margins of pronotum and elytra slightly paler.

Head. Head opisthognathous (Fig 1c, Supplementary Fig 1a, Supplementary Video 4), not visible in dorsal view and much narrower than overlying pronotum. Head vertex subtriangular, strongly transverse (Fig 1c, Supplementary Fig 1a, Supplementary Video 4), narrowing anteriorly (0.110 mm long, 0.272 mm wide; estimated HW/HL = 2.47);

antennae inserted under shelf-like margins of the frons (Fig 1d, Supplementary Video 4). Frons obtusely, triangularly protruding (Fig 1d, Supplementary Video 4). Labrum transverse, broadly emarginate medially, sparsely setose (Fig 1d, Supplementary Video 4). Eyes slender, mounted along margins of vertex/frons (Supplementary Figs 1a, b, Supplementary Video 4). Antenna thick, clavate and symmetrical; composed of eleven antennomeres, with apical eight antennomeres strongly clavate (Figs 1f, g, Supplementary Figs 1a, c, d, Supplementary Video 3). Antennomere I (a1) robust, thick, slightly elongate; a2 elongate, rather slender, dilated apically, as long as a1; a3 spherical, small, about 0.5 times as long as a2; a4 transverse, much broader but shorter than a3; a5 very transverse, slightly broader than a4; a6–10 extremely transverse, gradually widening towards apex, each almost same length; a11 elongate, subconical, large in size, as long as five preceding antennomeres. Pedicels of a4–a11 concealed or absent, basal connection of each antennomere hidden within apex of the antennomere more proximal so that nested antennomeres appear to telescope (Figs 1f, g, Supplementary Fig 1a, c, d, Supplementary Video 3). Surfaces of all antennomeres uniformly densely setose (Figs 1f, g, Supplementary Figs 1a, c, d, Supplementary Video 3). Maxillary palpomere (Fig 1e) relatively short, likely 4-segmented, palpomere 1 and basal portion of 2 not visible; palpomere 3 (mp3) long, slender, gradually widened apically, widest near apex, uniformly moderately pubescent; palpomere 4 (last; mp4) small, conical, much narrower than and approaching 1/3 length of palpomere 3 (Fig 1e, Supplementary Video 4). Mandibles falciform, without teeth (Fig 1d, Supplementary Fig 1a, Supplementary Video 4); left mandible lying over right mandible with bases closely juxtaposed (Fig 1d).

Pronotum. Broadly oval (Fig 1b), (0.440 mm wide, 0.250 mm long; width / length = 1.76), slightly narrower than elytra, widest slightly before base, with arcuate apical and posterior margins. Mesoscutellum fully concealed under pronotum and elytra (Fig 1b). Pronotal postcoxal process long, narrow, weakly pointed (Figs 1c, e). Mesosternal intercoxal process sharply pointed, with its apex lying slightly over that of metasternal intercoxal process (Supplementary Fig 1e, Supplementary Video 4); ventral surface expanded and shield-like (*sensu* Ashe, 2005[15]; see Kim *et al*. 2011[16], Fig. 10). Metaventrite subtrapezoidal (Fig 1c, Supplementary Video 2). Metasternal intercoxal process broadly rounded at apex (Supplementary Fig 1e, Supplementary Video 4).

Elytra and flight wings. Elytra (Figs 1a, b, 3a) subquadrate, short, only slightly longer than sutural length of pronotum (right elytron 0.289 mm long, left elytron 0.285 mm long, from posterior margin of pronotum to apex of elytron; right elytron 0.233 mm wide, left elytron 0.243 mm wide); weakly arcuate laterally, with posterolateral margins strongly sinuate; surface densely pubescent with fine microsetae, directed posterolaterally (Figs 1a, b, Supplementary Video 1). Hindwings not visible (Figs 1a, b, Supplementary Video 1).

Legs. Short, densely covered with microsetae (Figs 1a–c, Supplementary Fig 1f, Supplementary Videos 1, 2). Procoxae ovoidal, contiguous; profemora clavate, flattened (Supplementary Fig 1e), gradually narrowing apically, longer than procoxae; protibiae rather thick, only slightly widened toward apex, as long as or slightly shorter than profemora, lacking prominent spines laterally, with at least six long, unequally-sized setae at apex (Fig 1c, Supplementary Fig 1f, Supplementary Video 2); protarsi five-segmented, long, subequal in length to protibiae (Supplementary Fig 1f, Supplementary Video 2); tarsomere 1 (pt1) elongate, slightly longer than tarsomere 2, tarsomeres 2 and 3 subequal in length, tarsomere 4 slightly shorter than 3, tarsomere 5 as long as or slightly longer than tarsomeres 3 and 4 combined. Mesocoxae narrowly separated (Fig 1c, Supplementary Video 2); mesofemora clavate, narrowly elongate (Fig 1e, Supplementary Video 2); mesotibiae rather slender, only slightly widened toward apex slightly shorter than mesofemora, lacking prominent spines laterally, with approximately ten unequally-sized apical setae (Fig 1c, Supplementary Fig 1f, Supplementary Video 2); mesotarsi five-segmented, long, slightly longer than mesotibiae (Fig 1c, Supplementary Fig 1g, Supplementary Video 2); tarsomere 1 slender, long, slightly longer than tarsomeres 2 and 3 combined, tarsomere 2 slightly narrower than tarsomere 1, slightly longer than tarsomere 3, tarsomeres 3 and 4 in equal length, tarsomere 5 rather long, slightly longer than 4. Metacoxae contiguous, well developed with lamellae (Supplementary Figs 1g, g’, Supplementary Video 5); posterior lamella margin only slightly extends beyond posterior margin of coxa itself (see Naomi and Iwata, 1996[17] Figure 2E); metafemora clavate (Fig 1c, Supplementary 1f, Supplementary Video 2), narrowly elongate, longer than mesofemora; metatrochanters large, fusiform, basal portion partially concealed by lamella of metacoxa (Supplementary Figs 1g, g’, Supplementary Video 5); metatibiae slender (0.177 mm long), longer than mesotibiae, lacking prominent spines laterally (Fig

1c, Supplementary Fig 1f, Supplementary Video 2); metatarsi five-segmented, slender, long, moderately longer than metatibiae (Figs 1a–c, Supplementary Video 2); tarsomere 1 slender, long, nearly as long as next three segments combined; tarsomeres 2, 3, and 4 almost same length; tarsomere 5 moderately longer than preceding tarsomere. Claws simple and equal in size, arcuately curved (Supplementary Fig 1f). No clear empodial setae observed (Supplementary Fig 1f).

Abdomen. Six visible sternites, with partially visible sternite IX (Fig 1c, Supplementary Video 2). Shape triangular, wide, strongly and evenly tapering posteriorly, with six visible segments except terminalia (Figs 1a–c, Supplementary Videos 1, 2). Each segment of tergite/sternite widely transverse (Figs 1a–c, Supplementary Video 1, 2); segments III-VI more than three times wider than long; segments VII about two times wider than long. Tergites and sternites uniformly covered with dense, fine setae (Figs 1b–c, Supplementary Figs 1g, g', Supplementary Videos 1, 2). Tergites lacking pruinose spots (Fig 1b, Supplementary Video 1). Abdominal segments III–VII each with a pair of distinctly erect paratergites (Fig 1b, Supplementary Video 1). Tergite VII posterior margin simple, without any modification or emargination (Fig 1b, Supplementary Video 1). Tergite and sternite VIII generally rounded posteriorly (Figs 1b, c, Supplementary Video 1), but very weakly pointed medially, lacking long macrosetae. Sternite IX rather sharply pointed posteriorly (Fig 1c, Supplementary Video 1). Tergite X entire, pointed toward apex (Fig 1b, Supplementary Video 1).

Geographic and geological context. Burmese amber (burmite) is mined commercially, but extraction is confined to one locality: the Noije Bum mines in Hukawng Valley, Myitkyina district of Kachin state, northern Myanmar[18,19]. The amber has been traded for nearly 2,000 years, in particular with China, but its scientific importance was neglected until early this century[20] Ross *et al*. 2010. Burmese amber was initially thought to be of Cenozoic origin (Miocene-Eocene), but its Cretaceous age is now accepted and it has been dated to earliest Cenomanian (~99 Mya)[21]. The deposit is recognized as one of the most important from the Cretaceous[22]: the insect fauna was reviewed by Grimaldi *et al*. (2002)[20] and Ross *et al*. (2010)[23], with recent discoveries including a new fossil insect superorder, Pancondylognatha[24], two new orders, Alienoptera[25] and Permopsocida[26] and direct evidence of brood care in scale insects[27]. Beetles are diverse in Burmese amber[23],

with Staphylinidae comprising one of the predominant families: 20 species in 17 genera belonging to eleven staphylinid subfamilies have thus far been documented[14,28-39].

Systematic Position of *Mesosymbion*

Our first impression of *Mesosymbion* was of a limuloid trichopseniine with a similar body shape and specialized inquilinous head, but closer inspection revealed a lack of key autapomorphic features of trichopseniines, including the fusion of the metacoxae to the metaventrite. We compared *Mesosymbion* to several Recent and fossil taxa of Mesoporini (*Anacyptus*, *Dictyon*, *Kistnerium* and †*Palaeomesoporus*) and Trichopseniini (*Trichopsenius*, *Schedolimulus* and *Prorhinopsenius*), as well as detailed descriptions of other mesoporine and trichopseniine genera in the literature. *Mesosymbion* has all the characters that are consistent with the tribal diagnosis of Mesoporini: a small, limuloid body, head more or less concealed by pronotum, 5-5-5 tarsal formula, prominent reticulation on surface, mesoventral intercoxal process expanded like a shield, and a hindcoxal lamella. Currently, Mesoporini is comprised of ten genera (8 Recent): *Ambracyptus* Lundgren 1984 (fossil, Miocene Mexican amber); *Ampheida* Pace, 1990; *Anacyptus* Horn, 1877; *Dictyon* Fauvel, 1900 (including a fossil species from Eocene Baltic amber); *Kistnerium* Naomi & Iwata, 1996; *Mesoporus* Cameron, 1959; *Mimodictyon* Cameron, 1944; *Palaeomesoporus* Yamamoto & Maruyama, 2016 (fossil, Eocene Baltic amber); *Paraconosoma* Bernhauer, 1941; and *Paradictyon* Scheerpeltz, 1929. *Mesosymbion* is readily distinguished from all of these by its possession of thick, clavate antennae with compact antennomeres that are typical of many socially parasitic rove beetles. Other mesoporine genera have simple antennae with a loose to distinct club at the apex. *Mesosymbion* additionally has an opisthognathous head, while head orientation in the other genera of the tribe varies from prognathous to hypognathous; the head appears to be fully concealed under the pronotum (fully limuloid), while in other genera of Mesoporini the front of the head is partially exposed in dorsal view. The modified, shield-like head vertex, and the falciform shape and close juxtaposition of the mandibles are also unique. Despite its antiquity, *Mesosymbion* is arguably the most overtly morphologically specialized genus of its tribe.

Further resolution of the relationship between *Mesosymbion* and other mesoporine genera is not possible without a full phylogenetic analysis of the tribe, but we believe a position for *Mesosymbion* within the crown-group is likely due to its possession of all tribal characteristics (Supplementary Fig 2), and possible characters shared with certain crown-group genera. The new taxon may be closest to *Dictyon* and *Kistnerium* which have a similar maxillary palpal form[16,17], but in addition to lacking the unique traits of *Mesosymbion* mentioned above, these genera have non- or only weakly sinuate posterolateral margins of the elytra. Other mesoporine genera are readily separated from *Mesosymbion* by other character states. Three mesoporine genera have ten-segmented antennae (*Ampheida*, *Anacyptus*, *Mesoporus*). Furthermore, *Paraconosoma* and *Ambracyptus* have erect macrosetae on the abdomen and pronotum, respectively. *Mimodictyon* has a long maxillary palpomere 4 (mp4), half the length of palpomere 3 (mp3), while *Mesosymbion* has a much shorter mp4, nearly 1/3 the length of mp3. *Paradictyon* has a much larger body (1.6 mm), thicker maxillary palpomere 3, and lacks clearly sinuate elytral margins. Finally, *Palaeomesoporus* has much longer, slender, non-sinuate elytra, more strongly developed metacoxal lamella, and thicker mp4[10].

Supplementary References

1. Seevers, C. H. Fossil Staphylinidae in Tertiary Mexican amber (Coleoptera). *Univ. Calif. Publ. Entomol.* **63,** 77–86 (1971).
2. Lundgren, R. W. *Ambracyptus*, a new name for *Paracyptus* Seevers (Coleoptera: Staphylinidae). *Pan-Pac. Entomol.* **60,** 163 (1984).
3. Kistner, D. H. New species of termitophilous Trichopseniinae (Coleoptera: Staphylinidae) found with *Mastotermes darwiniensis* in Australia and in Dominican amber. *Sociobiology* **31,** 51–64 (1998).
4. Oustalet, E. Recherches sur les insectes fossiles des terrains Tertiaires de la France, deuxième partie, insectes fossiles d'Aix en Provence. *Annales des Sciences Géologiques* **5,** 1–347 (1874).
5. Semenov, V. B., Perkovsky, E. E. & Petrenko, A. A. The first finding of aleocharines (Coleoptera, Staphylinidae, Aleocharinae) from the Rovno amber. *Reports of the National Academy of Sciences Ukraine* 155–158 (2001).
6. Zerche, L. Eine neue Art der Gattung *Adinopsis* Cameron aus dem Baltischen Bernstein (Coleoptera: Staphylinidae, Aleocharinae, Deinopsini). *Beiträge zur Entomologie* **49,** 97–105 (1999).
7. Paśnik, G. & Kubisz, D. A new genus and new species of Staphylinidae (Coleoptera) from Baltic amber. *Eur. J. Entomol.* **99,** 353–361 (2002).
8. Paśnik, G. Fossils of Staphylinidae from Baltic amber: a new genus and three new species (Insecta, Coleoptera, Staphylinidae). *Seckenbergiana Biologica* **85,** 97–100 (2005).

9. Wolf-Schwenninger, K. A New Fossil Staphylinid Genus and Species from Baltic Amber (Coleoptera, Staphylinidae, Aleocharinae, Gymnusini). *Stuttgarter Beitrage zur Naturkunde Serie B Geologie und Palaontologie* **345,** 1–6 (2004).
10. Yamamoto, S. & Maruyama, M. A new genus and species of the rove beetle tribe Mesoporini from Baltic amber (Coleoptera: Staphylinidae: Aleocharinae). *Historical Biology* 1–5 (2016). doi:10.1080/08912963.2016.1144750
11. Wickham, H. F. Fossil Coleoptera from the Wilson Ranch Near Florissant, Colorado. *Bulletin from the Laboratories of Natural History of the State University of Iowa* **6,** 3–29 (1913).
12. Scudder, S. H. Fossil Coleoptera from the Rocky Mountain Tertiaries. *Bulletin of the United States Geological and Geographical Survey of the Territories* **2,** 77–87 (1876).
13. Scudder, S. H. *The Tertiary insects of North America.* 1–734 (Washington: Government Printing Office, 1890).
14. Cai, C. & Huang, D. The oldest aleocharine rove beetle (Coleoptera, Staphylinidae) in Cretaceous Burmese amber and its implications for the early evolution of the basal group of hyper-diverse Aleocharinae. *Gondwana Res.* **28,** 1579–1584 (2015).
15. Ashe, J. S. Phylogeny of the tachyporine group subfamilies and 'basal' lineages of the Aleocharinae (Coleoptera: Staphylinidae) based on larval and adult characteristics. *Syst. Entomol.* **30,** 3–37 (2005).
16. Kim, Y.-H., Lee, S.-G. & Ahn, K.-J. Description of *Dictyon termitophilum* New Species and a Key to the Species of the Genus *Dictyon* Fauvel (Coleoptera: Staphylinidae: Aleocharinae). *J. Kans. Entomol. Soc.* **84,** 174–178 (2011).
17. Naomi, S. & Iwata, R. *Kistnerium japonicum* Naomi and Iwata n. gen. and n. sp., the first representative of the Tribe Mesoporini (Coleoptera, Staphylinidae, Aleocharinae) from Japan, associated with Termites (Isoptera: Rhinotermitidae). *Sociobiology* **28,** 73–81 (1996).
18. Cruickshank, R. D. & Ko, K. Geology of an amber locality in the Hukawng Valley, Northern Myanmar. *J. Asian Earth Sci.* **21,** 441–455 (2003).
19. Kania, I., Wang, B. & Szwedo, J. *Dicranoptycha* Osten Sacken, 1860 (Diptera, Limoniidae) from the earliest Cenomanian Burmese amber. *Cretaceous Res.* **52,** 522–530 (2015).
20. Grimaldi, D. A., Engel, M. S. & Nascimbene, P. C. Fossiliferous Cretaceous amber from Myanmar (Burma): Its rediscovery, biotic diversity, and paleontological significance. *Am. Mus. Novit.* **3361,** 1–71 (2002).
21. Shi, G. *et al.* Age constraint on Burmese amber based on U-Pb dating of zircons. *Cretaceous Res.* **37,** 155–163 (2012).
22. Rasnitsyn, A. P. *et al.* Sequence and scale of changes in the terrestrial biota during the Cretaceous (based on materials from fossil resins). *Cretaceous Res.* **61,** 234–255 (2016).
23. Ross, A., Mellish, C. & Crighton, B. in *Biodiversity of Fossils in Amber from the Major World Deposits* 1–14 (Siri Scientific Press, 2010).
24. Yoshizawa, K. & Lienhard, C. Bridging the gap between chewing and sucking in the hemipteroid insects: new insights from Cretaceous amber. *Zootaxa* **4079,** 229–245 (2016).
25. Bai, M. *et al.* †Alienoptera — A new insect order in the roach–mantodean twilight

zone. *Gondwana Res.* (2016). doi:10.1016/j.gr.2016.02.002

26. Huang, D. *et al.* New fossil insect order Permopsocida elucidates major radiation and evolution of suction feeding in hemimetabolous insects (Hexapoda: Acercaria). *Sci. Rep.* **6,** 23004 (2016).
27. Wang, B. *et al.* Brood care in a 100-million-year-old scale insect. *Elife* **4,** e05447 (2015).
28. Chatzimanolis, S., Engel, M. S., Newton, A. F. & Grimaldi, D. A. New ant-like stone beetles in mid-Cretaceous amber from Myanmar (Coleoptera: Staphylinidae: Scydmaeninae). *Cretaceous Res.* **31,** 77–84 (2010).
29. Clarke, D. J. & Chatzimanolis, S. Antiquity and long-term morphological stasis in a group of rove beetles (Coleoptera: Staphylinidae): Description of the oldest *Octavius* species from Cretaceous Burmese amber and a review of the 'Euaesthetine subgroup' fossil record. *Cretaceous Res.* **30**, 1426–1434 (2009).
30. Yamamoto, S. The first fossil of dasycerine rove beetle (Coleoptera: Staphylinidae) from Upper Cretaceous Burmese amber: Phylogenetic implications for the omaliine group subfamilies. *Cretaceous Res.* **58,** 63–68 (2016).
31. Yamamoto, S. & Solodovnikov, A. The first fossil Megalopsidiinae (Coleoptera: Staphylinidae) from Upper Cretaceous Burmese amber and its potential for understanding basal relationships of rove beetles. *Cretaceous Res.* **59,** 140–146 (2016).
32. Parker, J. Emergence of a superradiation: pselaphine rove beetles in mid-Cretaceous amber from Myanmar and their evolutionary implications. *Syst. Entomol.* **41**, 541-466 (2016). doi:10.1111/syen.12173
33. Thayer, M. K., Newton, A. F. & Chatzimanolis, S. *Prosolierius*, a new mid-Cretaceous genus of Solieriinae (Coleoptera: Staphylinidae) with three new species from Burmese amber. *Cretaceous Res.* **34,** 124–134 (2012).
34. Jałoszyński, P., Yamamoto, S. & Takahashi, Y. *Scydmobisetia* gen. nov., the first definite Glandulariini from Upper Cretaceous Burmese amber (Coleoptera: Staphylinidae: Scydmaeninae). *Cretaceous Res.* **65,** 59–67 (2016).
35. Cai, C. & Huang, D. The oldest micropepline beetle from Cretaceous Burmese amber and its phylogenetic implications (Coleoptera: Staphylinidae). *Naturwissenschaften* **101,** 813–817 (2014).
36. Cai, C. & Huang, D. The oldest osoriine rove beetle from Cretaceous Burmese amber (Coleoptera: Staphylinidae). *Cretaceous Res.* **52,** 495–500 (2015).
37. Cai, C. & Huang, D. *Cretoleptochromus archaicus* gen. et sp. nov., a new genus of ant-like stone beetles in Upper Cretaceous Burmese amber (Coleoptera, Staphylinidae, Scydmaeninae). *Cretaceous Res.* **63,** 7–13 (2016).
38. Yamamoto, S. The oldest tachyporine rove beetle in amber (Coleoptera, Staphylinidae): A new genus and species from Upper Cretaceous Burmese amber. *Cretaceous Res*, 65: 163–171. (2016).
39. Lü, L., Cai, C., Huang, D. The earliest oxyteline rove beetle in amber and its systematic implications (Coleoptera: Staphylinidae: Oxytelinae). *Cretaceous Res.* (in press). (2016). http://dx.doi.org/10.1016/j.cretres.2016.09.008
40. von Beeren, C., Maruyama, M. & Kronauer, D. J. C. Cryptic diversity, high host specificity and reproductive synchronization in army ant-associated *Vatesus* beetles. *Mol. Ecol.* **25,** 990–1005 (2016).
